# Financial Health Literacy and the Shared Decision-Making Process in Healthcare

**DOI:** 10.3390/ijerph19116510

**Published:** 2022-05-27

**Authors:** Caitlin S. O’Mara, Justin P. Young, Zachary K. Winkelmann

**Affiliations:** 1 Department of Exercise Science, University of South Carolina, Columbia, SC 29208, USA; comara@email.sc.edu; 2 Department of Applied Medicine and Rehabilitation, Indiana State University, Terre Haute, IN 47803, USA; jyoung85@sycamores.indstate.edu

## 1. Patient-Centered Care

Patient-centered care is the delivery of care that is unique to each patient, individualized to their needs, and established through a shared patient–clinician decision-making process. Personal health literacy is the degree to which an individual can find, understand, and use information to make health-related decisions for themselves and others [1], and is critical to the delivery of patient-centered care. In the United States, more than 80 million Americans have a limited health literacy [2]. While they might seem simple to individuals with a health care background, the conversations happening in a doctor’s office may feel like communicating in a foreign language to others. Health literacy and the interpretation of medical information are skills often overlooked by physicians [3]. As a result, this assumed understanding significantly contributes to the lack of patient engagement and low-quality experiences happening during office visits. From an environmental standpoint, recent changes in the economy and healthcare models have resulted in greater financial responsibilities for patients [4]. To minimize financial burdens and achieve improved outcomes within healthcare, there needs to be an emphasis on patient engagement. Therefore, the purpose of this editorial is to introduce and discuss financial health literacy, identify common problems such as price transparency and financial responsibility, and provide recommendations on improving interprofessional shared decision making (SDM) as a central tenant to patient-centered care.

The National Academy of Medicine identified in 2001 that “all health professionals should be educated to deliver patient-centered care as members of an interdisciplinary team, emphasizing evidence-based practice, quality improvement approaches, and informatics” [5]. Specifically, patient-centered care is defined as delivering care specific to the patient and involving the individual and their beliefs in the shared decision-making process [6]. The delivery of patient-centered care has many benefits including decreased healthcare utilization and improved patient outcomes [7,8,9]. When healthcare providers use a patient-centered approach, the research suggests that it lowers healthcare costs as opposed to general medical care delivery [7,9]. According to the Institute for Patient- and Family-Centered Care (that houses the Picker Institute), there are four core components including dignity and respect, information sharing, participation, and collaboration [10]. However, the Institute of Medicine Committee on the Health Professions Education Summit report from 2003 goes beyond the four core components to include other specific skills that are provided in Table 1 [5].

In athletic training and sports medicine, researchers have identified that patient-centered care is one of the most frequently implemented core competencies among athletic training students [11,12]. There is also a large discrepancy in the reported percentage of patient encounters that involve patient-centered care [11,12,13]. This discrepancy suggests that athletic trainers may not fully understand the concept of patient-centered care or its implementation into practice, as they do not perceive that they are consistently implementing patient-centered behaviors across all patient encounters. However, most of this research has focused on the concepts of goal setting, clinician-rated outcome measures, and patient-reported outcome measures [11]. While goal setting and patient-reported outcome measures provide insight into aspects of patient’s beliefs and values and may improve SDM between a patient and clinician, they are not comprehensively representative of patient-centeredness. To date, there has been an unintentional under-representation of other domains related to patient-centeredness such as social determinants of health, patient advocacy, and health literacy in athletic training and sports medicine.

## 2. Shared Decision Making and Patient Engagement

Shared decision making (SDM) involves clinicians and patients working collaboratively to make decisions on treatment, testing, and direction of care [14]. Collaborative conversations are an essential part of patient-centered care because decisions are made with the patient’s preferences and values in the forefront. The American Hospital Association lists seven things patients gain when they are involved in SDM (Table 2). These benefits should be goals that all healthcare providers strive to achieve for their patients. Not only do they ensure that the patients receive the care they deserve, but they improve the provider’s ability to build clientele within their practice.

SDM and patient engagement have been shown to be positively related to one another, within the medical setting. The presence of shared decision-making significantly increases patient engagement [15]. In order to optimize patient engagement, the patient’s decision-making process must be understood. The Interprofessional SDM Model was developed to broaden the perspective of SDM and account for all significant individuals involved. This includes the patient, the family/surrogate/significant others, decision coach, and health care professionals [16]. The process of engaging in the SDM model allows for the patient to identify their goals and, more importantly, their choices surrounding their care. Through SDM, the patient, their family, and the health care team work collaboratively to develop these goals Once this understanding is gained, interventions that are individualized to the patient can be developed.

A key factor that needs to be addressed during the SDM process is the patient’s health literacy. The degree to which the patient is involved in the process is highly influenced by their level of health literacy [16]. There are three levels of health literacy: functional, communicative/interactive, and critical. In a recent survey conducted in the United States, only 18% of healthcare consumers were rated as having the highest level of health literacy [17]. The level of health literacy that an individual holds can change over time and is influenced by many factors. Culture, race, gender, education, age, and socioeconomic status are all key social determinants that should be taken into consideration [18].

## 3. What Is Financial Health Literacy?

Financial health literacy is one’s ability to access, understand, and utilize financial health information to achieve improved health and financial outcomes [19]. Simplified, financial health literacy is the ability of an individual to make sense of the costs associated with their healthcare, such as medical bills or prescription medications. In a 2019 report from Waystar, 38% of healthcare consumers stated that they did not know that the cost of their healthcare varied across different facilities [20]. Patients are often unaware of this information and are not equipped with the knowledge or skills to access it, as there is no designated resource that is responsible to assist them in navigating or accessing this information. Some individuals believe that it is the doctor’s responsibility, but that belief is not equally shared [20]. Based on a recent study published in the Annals of Internal Medicine, few clinicians are even discussing cost with their patients [20]. These conversations happen in only 46% of patient encounters [21].

One example in which financial health literacy is a key consideration is the use of diagnostic imaging for musculoskeletal injuries [3,22]. As the price of healthcare expenditures climb, the overuse of diagnostic imaging, specifically magnetic resonance imaging (MRI), has become a significant contributor to patients’ out-of-pocket costs [23]. Considering that a small percentage of patients have a high health literacy level [16], and few clinicians are discussing the cost of care with their patients [20], a substantial portion of patients are vulnerable to accepting serious financial responsibility due to the costs associated with diagnostic imaging. In a study observing the overuse of MRI in the treatment of moderate to severe osteoarthritis, it was concluded that there is a need for improved education amongst non-physicians and non-academic physicians [3]. In general, MRI use in the evaluation of end-stage knee joint osteoarthritis is unnecessary, specifically when there is pre-existing supporting evidence from X-rays or clinical examination. Up to 45% of knee MRIs ordered by physicians outside of the orthopedic specialty were interpreted as normal, aside from pre-existing osteoarthritis [22]. In many of these cases, the MRI was useless. It provided no new or helpful information to the patient or provider. It simply contributed to the cost of care [22]. Similar findings have been observed in the treatment of non-specific hip pain. Cost utility is a method used to determine the effectiveness of the intervention, such as an MRI [24]. It has been revealed that MRI cost utility exceeded 11,000 USD when ordered by a non-orthopedic specialist [25]. This is more than three times as expensive when compared to an MRI study being ordered by an orthopedic surgeon [24]. Collectively, providers need to assess whether the value of their decisions justifies the associated expenses. To avoid unnecessary imaging, the literature suggests the creation and implementation of clinical practice guidelines. The use of clinical practice guidelines and similar resources will lessen the number of unwarranted MRIs and other diagnostic imagining, thereby decreasing the financial burden on patients.

## 4. Improving Financial Health Literacy

In order to increase the frequency and effectiveness of collaborative conversations, there needs to be a designated group initiating them. One solution is hiring or developing patient navigation staff. Similar to a department of financial services, this individual can be located within each department and can assist patients in gaining a better understanding of what they are being charged for, what their insurance covers, and when their bill is expected to be paid. This idea of price transparency should be a top priority for medical organizations. Recently, strides have been made within the medical community towards the improved price transparency of costs associated with health care. On 1 January 2021, the Centers for Medicare & Medicaid Services passed the Hospital Price Transparency rule [26]. Under this rule, each hospital in the United States is required to provide clear and accessible pricing information on their website. Previous research has shown that few patients utilize price transparency tools [27]. Having chargemaster prices listed in a consumer-friendly format allows the patients to “price shop” and estimate the cost of their care before going to the hospital. Having this resource in place can save a patient from unnecessary out-of-pocket expenses and frustrations as a result of their visit.

In sports medicine, we recommend the use of an athletic trainer to navigate patient-centered care domains when working on collaborative care. Athletic trainers can help patients succeed by preparing them for the conversations and decisions that may occur in physician clinics and other healthcare settings. If a patient is aware of questions that will be asked, the decisions that may be made, and the potential costs and impacts of those decisions, they may have the confidence necessary to be an active participant in the SDM process. As an athletic trainer in the physician practice setting, there may be opportunities for greater involvement as part of a patient support team. Previous research has suggested several strategies to facilitate SDM. Patient decision aids, decision coaching, and question prompt lists have all been indicated to improve patient knowledge [16].

From the physician perspective, there is conflicting evidence on the level of involvement that is necessary [2,3]. Some believe that physicians are trained to care for the patient in front of them, and not to assume responsibility for entire populations [28]. However, in a time where medical bills are the leading cause of personal bankruptcy [29], there are ethical standards that should be upheld. The shift to value-based healthcare has resulted in patients demanding price transparency and cost-effective treatment. To maintain the consistency and progress of these standards, there needs to be formal training. Costs of Care is one example of how a clinician may obtain formal training in this area. Costs of Care is a nonprofit organization that provides a resource for that type of training [30]. Their goal is to educate medical professionals on the finances of healthcare and how to save their patients from financial burdens. Implementing educational programs like this is not meant to add complexity to the jobs of providers. Instead, they are there to elevate the level of care for the patient.

## 5. Advancing Your Patient-Centered Care

The financial expectations associated with health care delivery create a dynamic process that constantly evolves. Best practices should not be reserved for medical evaluations and treatment. To be patient-centered and embody the principles of patient-centeredness established by the National Academy of Medicine and the Institute for Patient- and Family-Centered Care, a provider must consider patients’ individual needs and preferences when providing care options for SDM which includes a patient’s financial health literacy. An individual’s financial health should be considered in every encounter, and opportunities to better educate patients on financial health should be prioritized. The SDM process should include discussion on the potential financial costs of care decisions and incorporate financial planning as a part of care planning. It would be a disservice to the patient community if their providers did not maintain a current understanding of the financial trends and needs associated with those evolutions. Physicians, athletic trainers, and other medical professionals should seek continuing education on the subject, to provide their patients with the best possible resources. Additionally, providers should push for administrative assistance in policy development and implementation surrounding financial support for patients.

## Figures and Tables

**Table 1:** Skills of Patient-Centered Care.

Patient-Centered Care Key Component	Specific Skills
Share power and responsibility with patients and caregivers.	Discuss short and long-term goals specific to both their healthcare visit and return to work, life, and sport Acknowledging those around you that may have a role or responsibility in the care and creating support systems Promote self-care Managing one’s pain, comfort, assistance with living, and reducing fears or anxiety relative to health and healthcare Respect Discuss time available before starting the interaction
Communicate with patients in a shared and fully open manner.	Coordinate and integrate care Promote team-based care with multiple people and providers Address health literacy for each individual and their support system
Consider patients’ individuality, emotional needs, values, and life issues.	Address each person’s social determinants of health Consider how marginalized communities may experience health and healthcare differently Integrate whole-person, holistic healthcare
Advocate and reach patient, caretakers, and other social support systems in the community	Use person-first language when addressing or discussing a patient Summarize information gathered often and advocate for clear understanding before continuing with the examination
Enhance prevention and health promotion	Inform others on the process of care including injury and illness prevention Promote and optimize wellness in all dimensions of health

**Table 2:** Patient Benefits from Shared Decision Making.

Gain a better understanding of their health and health conditions
Decision making recognition with informed options
Understanding of pros and cons
Obtain tools needed to evaluate their options
Preparation for talking with healthcare providers
Collaboration opportunities with their healthcare team
Higher likelihood of following through on their decisions
